# A qualitative review of the design thinking framework in health professions education

**DOI:** 10.1186/s12909-019-1528-8

**Published:** 2019-04-04

**Authors:** Jacqueline E. McLaughlin, Michael D. Wolcott, Devin Hubbard, Kelly Umstead, Traci R. Rider

**Affiliations:** 1 UNC Eshelman School of Pharmacy, UNC Chapel Hill, Chapel Hill, North Carolina USA; 2 UNC Adams School of Dentistry, UNC Chapel Hill, Chapel Hill, North Carolina USA; 3 UNC School of Medicine and UNC/NC State Joint Department of Biomedical Engineering, UNC Chapel Hill, Chapel Hill, North Carolina USA; 4 NC State University College of Design, Raleigh, North Carolina USA; 5 Design Initiative for Sustainability and Health, NC State University College of Design, Raleigh, North Carolina USA; 6 Center for Innovative Pharmacy Education and Research, UNC Chapel Hill, Chapel Hill, North Carolina USA

**Keywords:** Design thinking, Curriculum development, Creativity, Problem solving, Innovation

## Abstract

**Background:**

Design thinking is a problem-solving framework that has been used to enhance patient experiences, improve clinical outcomes, and refine medical curricula. This study reviewed the use of design thinking in health professions education.

**Methods:**

A search yielded 169 articles, which were excluded if they were: (1) not related to education; (2) lacking an application of design thinking; or (3) not associated with healthcare. The final review yielded 15 articles, which were analyzed using qualitative methods.

**Results:**

All articles were published in 2009 or later and were diverse in their context, participants, and approach. Six studies emphasized the early stages of design thinking, with inspiration and ideation stages fostered through a variety of activities, such as lectures, small group discussions, and workshops. Studies examined a range of outcomes, including self-efficacy, perceptions, and solutions to a specific problem.

**Conclusions:**

Our findings raise important considerations for health professions education, including the extent to which we should: 1) teach design thinking to students as a skill-based tool to prepare students for problem solving in complex healthcare environments; and 2) use design thinking to create, implement, and refine health professions curricula and educational programs. Despite the apparent benefits of design thinking, many questions for health professions education remain.

**Electronic supplementary material:**

The online version of this article (10.1186/s12909-019-1528-8) contains supplementary material, which is available to authorized users.

## Background

A growing body of literature highlights the increasing demand on healthcare providers to simultaneously master the clinical content and thinking processes necessary to address complex patient-care problems, particularly in the face of uncertainty within a dynamic and rapidly evolving environment [1–5]. The demand for these diverse and adaptable problem solving skills has led to a vast and disparate body of research with a wide range of proposed solutions [6–8]. Design thinking is one framework for complex problem solving applied widely by various disciplines and recently emerging within healthcare [9–21].

As a methodology, the origin of design thinking is often credited to Herbert A. Simon’s Sciences of the Artificial in 1969 [22]. As a term, design thinking is often traced to Bruce Archer (1979), who stated *there exists a designerly way of thinking and communicating that is both different from scientific and scholarly ways of thinking and communication, and as powerful as scientific and scholarly methods of inquiry* [23]. Since then, design scholars have been influential across various areas intersecting the professions. Donald Schön’s work on the reflective practitioner, for example, emerged from his portrayal of design as a technical-rational process [24]. In addition, Jornet and Roth reframed design as intrinsically social and Krippendorff emphasized the communicative nature of design, both of which help frame the ways in which health professionals can engage in the design process to solve problems with and for patients [25, 26].

The design process recently popularized by Tim Brown describes three stages in the design thinking cycle: 1) *inspiration*, which embodies the initial problem or opportunity; 2) *ideation*, which encompasses the development and refinement of ideas; and 3) *implementation*, which involves the introduction and application of the derived solution [27]. Other organizations and disciplines (e.g. biodesign, design-based research) have presented design thinking as an expanded five stage process such as: (1) *empathy* or *discovery*, where the goal is to understand the audience for who you are designing (2) *define* or *interpretation*, which involves describing the point of view and needs of the individual, (3) *ideate* or *ideation*, that includes brainstorming to produce as many creative solutions as possible, (4) *prototype* or *experimentation*, where a potential solution is crafted to be able to manipulate and identify flaws, and (5) *test* or *evolution,* which includes sharing the protoype with the target users to obtain feedback and lead to modifications.

While variations of the design thinking structure (and nomenclature) exist [28], all allow for bidirectional movement to and from each step as a problem is addressed [29]. This flexible process encourages iterative exploration of solutions, continual refinement of the problem space, and increased understanding of user needs. Cognitive characteristics of design thinking include open-mindedness, suspension of judgement, and a bias toward action. Within a team setting, diversity and collaboration across disciplines, viewpoints, and backgrounds are highly valued and are generally required for successful problem solving [30]. Together, the process and mindset of design thinking present a unique framework for problem solving that could benefit aspiring health professionals faced with complex clinical decision making.

Design thinking has recently been applied in healthcare to address patient experiences, clinical outcomes, and health care spending [9, 10, 31–33]. Literature also indicates that medical educators are integrating design thinking into their curricula [15, 34–37], and that medical schools are partnering with design firms to better understand behavior in clinical settings [34]. This design focus aligns with the work of Manzini, which extends design beyond mainstream product development to social innovation and sustainability that more closely mirrors design needs in service professions, such as healthcare and education [38]. The small but growing relationship between medical education and the design thinking framework provides a timely opportunity to assess research in this area and identify promising opportunities. The purpose of this review was to examine the use of design thinking in health professions education with the primary aim of understanding how design thinking has been applied and assessed. This is the first review to examine this topic.

## Methods

Since this review aimed to provide a descriptive overview of a diverse body of literature pertaining to a broad topic, we employed a scoping review methodology: identify the study aim; identify relevant studies; select studies for inclusion; analyze studies; collate, summarize, and report the results. First, we identified our overarching aim, which was to examine the use of the design thinking in research on health professions education. This aim included understanding both the nature of design thinking discourse (e.g. was design thinking centrally integrated throughout the work?), and the components, results, and benefits of design thinking.

Then, we indentified relevant studies by conducting a broad, preliminary search by searching MEDLINE, Embase, CINAHL, JSTOR, Web of Science, Science Direct, ERIC, and PsychInfo for “design thinking” and related synonyms, such as “design cognition”, “design behavior”, “prototyping”, “divergent thinking”, and “biodesign”. The synonyms offered little benefit beyond use of “design thinking” and were dropped from subsequent searches. To better scope the review, a hybrid search strategy was identified in consultation with a university librarian. For health-related databases, the search term “design thinking” was used alone, whereas in education- and science-related databases additional search terms included a list of healthcare terms (Additional file 1). Of note, this study focused explicitly on “design thinking” and excluded design-related articles that did not explicitly incorporate the term “design thinking,” which means that some literature associated with instructional design, instructional systems development, and learning design were purposefully excluded if it did not explicate design thinking.

**Fig. 1:**
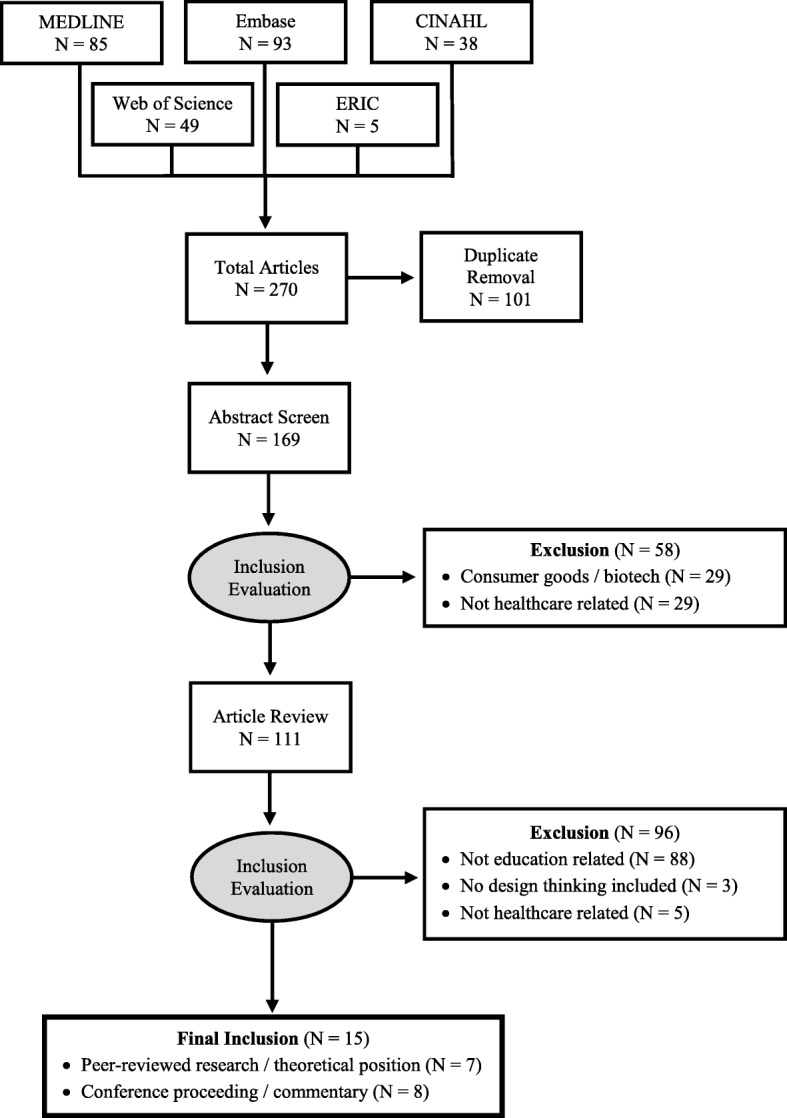
Article Review Flow Diagram

The search for studies was conducted in February 2018 and returned 169 citations with a starting point of 1979, which was when design thinking terminology emerged [23]. The abstracts of all citations were reviewed independently by two members of the research team (JM and MW). The goal was to identify research at the intersection of design thinking and health professions education; in other words, we searched for articles that described design thinking strategies married with pedagogy or educational programs/curricula in healthcare. In the abstract review, 111 articles were selected to advance by at least one reviewer. We excluded reviewed articles in a hierarchical fashion if they were: (1) not related to education (*n* = 88); (2) lacking an application of design thinking (e.g. design thinking only listed in references or author position titles) (*n* = 3); or (3) not associated with healthcare (*n* = 5). Initial agreement was 100% for inclusion. The final review resulted in 15 articles fitting the inclusion criteria (Fig. 1) [15, 36, 39–43].

The review was conducted in two phases. In the first phase, the aim was to characterize the nature of discourse in the article. Using a *theory talk* coding schema adapted from Kumasi and colleagues [44, 45], the articles were coded as major theory talk, moderate theory talk, or minimal theory talk. Articles using major theory talk were those that used design thinking as a core element of the study, for example to inform design and data analysis, test an existing theory or instrument, and/or create new theory. Moderate theory talk involved conference proceedings, commentaries, and editorials. Minimal theory talk provided little to no reference to design thinking or its related constructs. This coding scheme enabled us to focus on articles in which design thinking was a core element of the work.

In the second phase, we conducted a qualitative review of the articles characterized by *major theory talk* [44]. Qualitative analysis was used because it focuses on the language and contextual meaning of texts [46, 47]. The findings were organized and summarized according to three questions: 1) What model or model components of design thinking are emphasized in health professions education? 2) What results are examined in this literature? and 3) What benefits of design thinking do researchers identify for health professions education?

## Results

Agreement by two reviewers for theory talk category was 100%. Of note, no articles qualified as minimal theory, 8 used moderate theory talk (e.g. commentaries, conference proceedings), and 7 were identified as major. All major theory talk articles were published between 2009 and 2017 (Table 1). Research was conducted in the United States (*n* = 4), United Kingdom (*n* = 1), Denmark (*n* = 1), and Netherlands (*n* = 1). Most articles included medicine (*n* = 7), while 4 engaged other professions, such as nursing, pharmacy, physiotherapy, occupational therapy, art, and engineering. Participants were primarily practitioners (*n* = 4), with research also including students (*n* = 3), program directors (*n* = 1), and patients (*n* = 1).

**Table 1:** Description of articles included in the final review examining the use of design thinking in health professions education

Publication date	Program/Aim	Disciplines Included	Study Location	Participants	DT Model Used	Outcome(s) Described	Examples of Benefits of DT Identified
2009	Educate intensivists to use design thinking to marshal support for adopting technology	Medicine	United States	Practitioners, administrators	Brown (3-stage)	Acquisition of technology solutions	Understanding user needs; iterative prototyping to refine approach and decision making
2014	Teach design thinking to inpatient spinal cord injury patients using a series of 4 to 5 workshops	Medicine	United Kingdom	Patients	Brown (3-stage)	Patient self-efficacy and perceptions, PMnac, ADAPSS, PAM, EQ-5D, HRQL, length of stay, readmission rate	Improved outcomes; interdisciplinary collaboration; flexibility to improve and align program with context
2016	Conduct a 2-day competitive interdisciplinary team event (i.e. “hackathon”)	Medicine, engineering, design, business	United States	Students, practitioners, scientists, engineers	Not Defined	Demographics	Interdisciplinary collaboration
2016	Design a 2-day seminar aimed at generating interprofessional education (IPE) evaluation strategies	Medicine, nursing, pharmacy, physician assistant	United States	Practitioners	Modified (5-stage)	Model for evaluating IPE	Flexible process
2016	Design and implement a semester-long “Hacking Healthcare” course	Medicine, social sciences, and art	Netherlands	Students	Brown (3-stage)	Student project solutions; student perceptions	Activating environment; interdisciplinary collaboration; empathetic “design min-set”
2017	Investigate camp designed to provide hands-on experience in creativity, innovation, and entrepreneurship	Physiotherapy, occupational therapy, radiography, nursing, and midwifery	Denmark	Students	Brown (3-stage)	Perceived relevance of camp, format, and effort	Interdisciplinary cooperation; real-world/relevant problems; active learning environment
2017	Characterize innovation and entrepreneurship programs in US medical education	Medicine	United States	Program Directors	Not Defined	Characteristics of innovation and entrepreneurship programs	Advancing readiness for complex problems; user-centric; active; interdisciplinary

*DT* = Design Thinking

### What model or model components of design thinking are emphasized in health professions education?

Five articles cited the design thinking model defined by Brown [24], with three of those articles explicating the design stages used. Some variations appeared, with one study using 1) discovery; 2) interpretation; 3) ideation; 4) experimentation; and 5) evolution. One article did not include a design thinking reference, instead providing a general description of the design thinking process. Another article provided a review of innovation and entrepreneurship programs and did not explicate a specific design thinking framework.

Design thinking was used in 6 studies as a methodology for designing educational strategies or programs (Table 1). Only 2 studies explicitly taught the subject of design thinking to participants as a tool for problem solving; in four articles, participants were led through the design thinking process, however the extent to which the researchers explicated the process to participants was unclear. Six studies emphasized the early stages of design thinking, with inspiration and ideation stages fostered through a variety of activities, such as lectures, small group discussions, and workshops. Only two studies appeared to engage participants in later stages of the process (i.e. implementation). Wolstenholme and colleagues, for example, highlighted the iterative process of their work with patients, which included multiple rounds of evaluation and change to program structure [39]. Van de Grift and Kroeze had students explicate the implementation process and presented the final prototype to pertinent stakeholders [33].

### What outcomes are examined in this literature?

The reviewed studies employed various study designs and subsequently reported a wide range of outcomes. Four articles used mixed methods, 2 reported only qualitative findings, and 1 provided only quantitative results. Five articles described immediate outcomes, 1 provided longitudinal data, and 1 reported descriptive and thematic characteristics of innovation and entrepeneurship programs. It may be worth noting that none of the reported outcomes measured the design thinking cycle or any of its individual components; rather, the researchers used design thinking as a methodology to produce and examine other outcomes. Those outcomes included self-efficacy and confidence, participant experiences, program characteristics, and solutions to a specific problem (e.g. interprofessional education evaluation framework). Van de Grift and colleagues, for example, reported positive student experiences in an interdisciplinary *Healthcare Hacking* course attributed to the activating teaching environment, development of collaboration skills, and academic development [36].

### What benefits of design thinking do researchers identify for health professions education?

Despite the wide-range of outcomes and contexts examined in this literature, researchers generally converged on the benefits associated with using design thinking in health professions education. All highlighted the importance and benefit of collaboration, particularly as it related to the multidisciplinary teams and the diversity of thinking that advanced the work as well as the identification and participation of multiple stakeholders within the process. Several acknowledged the value of providing opportunities to develop interdisciplinary communication skills through collaborative design processes.

Design thinking was credited with helping participants refine problems and identify the appropriate needs with a human- or user-centered approach. As noted by van de Grift and colleagues, *Outcomes of the case studies were generally both practical and unexpected, which was made possible by the methodology of iterative human-centered design thinking* (p. 1237) [36]. Rethinking the problem-solving process as learner-centered was largely offered as an approach that could advance creativity and communication skill development, improve patient outcomes, and enhance practice models. Several studies further emphasized the importance of context in the problem-solving process and acknowledged the flexibility of design thinking as a benefit for adapting the model to various time scales, industries, and circumstances.

## Discussion

This review explored the use of design thinking in research on health professions education as a first step toward understanding how design thinking has been applied in health professions training and education. Given its growing use in other disciplines (e.g., business, service design, and social policy) and increasing popularity in healthcare, we were surprised to uncover such a small body of literature. However, this appears to be an emerging research front, as evidenced by: [1] the contemporary nature of this topic, with all reviewed articles dating 2009 or later; and [2] the level of academic discourse, with more than half of the included articles coded as editorials, commentaries, or other *moderate theory talk* works.

This review indicates that educators are using design thinking as a tool and topic in the education and training of practitioners, patients, and students. In *Doctors as Makers,* Baruch implores medical curricula to foster creative and critical thinkers that can work through not-knowing, seek compassionate solutions, and explore questions differently within an environment supportive of iterative development [1]. Design thinking is well-suited to meet these needs, as the concepts, skills, and mindset of design thinking can foster solutions to ill-defined, complex, and unusual problems. Farrell & Hooker highlight the importance of design and science for addressing these types of *wicked problems*, stating that “…design method, like scientific research method, is a product of a common core cognitive process and management of pragmatic complicating conditions” [48]. These apparent benefits, along with the emphasis on science and design, may explain the growing number of medical education programs incorporating the field of design thinking in its instruction [15, 34–36, 49].

In addition to equipping individuals with design thinking skills, this review suggests that design thinking can be used to inform curricula and programs (e.g., hackathons, workshops), shape organizational processes, and redesign curricula. Considering education as a user-centered product/service and approaching curriculum development as a design challenge could elucidate novel solutions to the many challenges facing health professions education. Harvard Medical School, for example, recently described a student-centered design approach to inform pedagogical changes [49], while Badwan and colleagues posited the use of design thinking for the development and implementation of teaching and learning technologies in medical education [50].

As noted by Simon (1996), *Everyone designs who devises courses of action aimed at changing existing situations into preferred ones. The intellectual activity that produces material artifacts is no different fundamentally from the one that prescribes remedies for a sick patient…Design, so construed, is the core of all professional training…(p. 111)* [22]. Clearly, there are opportunities for exploring the intersectionality of design thinking and health professionals education. The small yet contemporary body of literature in this review gives way to numerous questions that could help elucidate this further. For example, in what ways does design thinking align with other problem-solving processes in health professions training (e.g. clinical decision-making)? How can design thinking be used to develop empathy and other patient care skills in aspiring healthcare professionals? What strategies are best suited for integrating design thinking into our curricula – is it a standalone course, a subtopic integrated into other courses, a hackathon, or something else? What criteria should we use to evaluate the quality and impact of design thinking (e.g., developing design thinking metrics)? [51, 52]. These questions, among others, highlight the need and opportunity for further research that elucidates the potential role of this technique in healthcare training and practice.

This study has several limitations. The databases used in this search were domain specific. While it is possible that our review missed some relevant articles, we used diverse databases from education and health professions to minimize this risk. Also, the review focused on work using the phrase “design thinking”, which may have excluded articles with related terms or individual elements of the design thinking process (e.g. empathy, prototyping). This focus also purposfully excluded literature that did not explicate “design thinking,” which may have exluded literature associated with instructional design, instructional systems design, and learning design. Despite these limitations, this review provides insight into the use of design thinking to-date, highlights the potential benefits of this model, and informs the development of a design thinking research agenda for health professions education. Further research must be conducted to better understand the impact and utility of design thinking for the health professions, determine the best model for engendering this model to learners, and identify outcomes to measure that will elucidate the effectiveness of our work.

## Conclusions

This study revealed a small yet contemporary body of literature associated with design thinking and health professions education. We hope this review further promotes design thinking efforts to identify promising solutions for developing problem-solving skills in aspiring and practicing health professionals, creating programs and curricula that meet our educational needs, and improving patient care amid an increasingly complex healthcare system.

## Additional file

Additional file 1: Search Strategy for ERIC and Web of Science. List of terms used for conducting literature search. (DOCX 23 kb)
