# Supplementary material for: A qualitative review of the design thinking framework in health professions education
Source: BMC Med Educ. 2019 Apr 4;19:98. doi: 10.1186/s12909-019-1528-8 (PMC6449899; doi:10.1186/s12909-019-1528-8)
Supplement: Supplementary file 1 — Search Strategy for ERIC and Web of Science. List of terms used for conducting literature search. (DOCX 23 kb) [file 12909_2019_1528_MOESM1_ESM.docx]

**Appendix A**. Search Strategy for ERIC and Web of Science

| healthcare OR "health care" OR “Allied Health Professional” OR “Allied Health Professionals” OR “Population Program Specialists” OR “Population Program Specialist” OR “Healthcare Assistants” OR “Healthcare Assistant” OR “Healthcare Support Workers” OR “Healthcare Support Worker” OR “Paramedics” OR “Paramedic” OR “Paramedical Personnel” OR “Animal Technician” OR “Animal Care Technicians” OR “Animal Care Technician” OR “Veterinary Technician” OR “Veterinary Technicians” OR “Veterinary Nurses” OR “Veterinary Assistants” OR “Veterinary Nurse” OR “Animal Care Assistants” OR “Animal Care Assistant” OR “Veterinary Assistant” OR “Laboratory Animal Technologists” OR “Laboratory Animal Technologist” OR “Community Health Worker” OR “Community Health Aides” OR “Community Health Aide” OR “Family Planning Personnel” OR “Village Health Workers” OR “Village Health Worker” OR “Barefoot Doctors” OR “Barefoot Doctor” OR “Family Planning Personnel Characteristics” OR “Dental Auxiliary” OR “Dental Receptionist” OR “Dental Receptionists” OR “Dental Nurses” OR “Dental Nurse” OR “Dental Assistant” OR “Dental Hygienist” OR “Dental Technician” OR “Denturist” OR “Denturism” OR “Emergency Paramedic” OR “Emergency Paramedics” OR “Emergency Medicine Technicians” OR “Emergency Medicine Technician” OR “Emergency Medical Technician” OR “Home Health Aide” OR “Homemaker-Home Health Aides” OR “Homemaker Home Health Aides” OR “Homemaker-Home Health Aide” OR “Home Care Aides” OR “Home Care Aide” OR “Licensed Practical Nurse” OR “Licensed Vocational Nurses” OR “Licensed Vocational Nurse” OR “Medical Record Administrator” OR “Medical Record Librarians” OR “Medical Record Librarian” OR “Medical Record Technicians” OR “Medical Record Technician” OR “Medical Secretary” OR “Medical Receptionist” OR “Nurse Aides” OR “Nurses Aides” OR “Nurse s Aide” OR “Nursing Auxiliaries” OR “Nursing Auxiliary” OR “Psychiatric Aide” OR “Psychiatric Hospital Orderlies” OR “Psychiatric Hospital Orderly” OR “Psychiatric Ward Attendants” OR “Psychiatric Ward Attendant” OR “Operating Room Technician” OR “Pharmacy Technician” OR “Pharmacists Aides” OR “Pharmacist Aides” OR “Pharmacist s Aides” OR “Pharmacists Aide” OR “Physical Therapist Assistant” OR “Physical Therapy Assistants” OR “Physical Therapy Assistant” OR “Physician Assistant” OR “Physicians Extenders” OR “Physician Extenders” OR “Physician s Extenders” OR “Physicians Extender” OR “Doctor s Assistants” OR “Doctor Assistants” OR “Doctors Assistant” OR “Physicians Assistants” OR “Physician s Assistants” OR “Physicians Assistant” OR “Feldshers” OR “Feldsher” OR “Ophthalmic Assistant” OR “Pediatric Assistant” OR “Anatomist” OR “Anesthetist” OR “Anesthesia Assistants” OR “Anesthesia Assistant” OR “Anesthesiologist” OR “Physician Anesthetists” OR “Physician Anesthetist” OR “Nurse Anesthetist” OR “Audiologist” OR “Caregiver” OR “Carers” OR “Carer” OR “Care Givers” OR “Care Giver” OR “Spouse Caregivers” OR “Spouse Caregiver” OR “Family Caregivers” OR “Family Caregiver” OR “Case Manager” OR “Medical Examiners and Coroners” OR “Coroners” OR “Coroner” OR “Medical Examiner” OR “Medical Examiners” OR “Dental Staffs” OR “Hospital Dental Staffs” OR “Hospital Dental Staff” OR “Dentist” OR “Prosthodontists” OR “Prosthodontist” OR “Prosthetic Dentist” OR “Prosthetic Dentists” OR “Restorative Dentist” OR “Restorative Dentists” OR “Pediatric Dentist” OR “Pediatric Dentists” OR “Periodontists” OR “Periodontist” OR “Women Dentists” OR “Woman Dentist” OR “Woman Dentists” OR “Endodontist” OR “Maxillofacial Surgeons” OR “Maxillofacial Surgeon” OR “Oral Surgeons” OR “Oral Surgeon” OR “Exodontists” OR “Exodontist” OR “Orthodontist” OR “Dentofacial Orthopedists” OR “Dentofacial Orthopedist” OR “Doula” OR “Labor Coaches” OR “Labor Coach” OR “Emergency Medical Dispatchers” OR “Epidemiologist” OR “Dental Faculties” OR “Dental Faculty” OR “Medical Faculty” OR “Medical Faculties” OR “Nursing Faculty” OR “Nursing Faculties” OR “Health Educator” OR “Health Facility Administrator” OR “Hospital Administrator” OR “Hospital CEO” OR “Hospital Chief Executive Officer” OR “Hospital Chief Executive Officers” OR “Infection Control Practitioner” OR “Medical Chaperone” OR “Patient Chaperones” OR “Patient Chaperone” OR “Medical Laboratory Personnels” OR “Clinical Laboratory Personnel” OR “Clinical Laboratory Personnels” OR “Medical Laboratory Scientists” OR “Medical Laboratory Scientist” OR “Medical Technologists” OR “Medical Technologist” OR “Clinical Laboratory Scientists” OR “Clinical Laboratory Scientist” OR “Medical Laboratory Technicians” OR “Medical Laboratory Technician” OR “Clinical Laboratory Technicians” OR “Clinical Laboratory Technician” OR “Medical Laboratory Assistants” OR “Medical Laboratory Assistant” OR “Medical Staffs” OR “Hospital Medical Staff” OR “Hospital Medical Staffs” OR “Junior Physician” OR “Junior Physicians” OR “Hospital Registrar” OR “Hospital Registrars” OR “Hospital Attending Physicians” OR “Hospital Attending Physician” OR “Hospitalist” OR “Nurse” OR “Nursing Personnel” OR “Registered Nurses” OR “Registered Nurse” OR “Nurse Administrator” OR “Nurse Managers” OR “Nurse Manager” OR “Nurse Executives” OR “Nurse Executive” OR “Nurse Practitioner” OR “Family Nurse Practitioner” OR “Pediatric Nurse Practitioner” OR “Nurse Specialist” OR “Nurse Clinician” OR “Clinical Nurse Specialists” OR “Clinical Nurse Specialist” OR “Nurse-Midwife” OR “Nurse Midwife” OR “Nurse-Midwives” OR “Pediatric Nurse” OR “Pediatric Nurses” OR “Neonatal Nurse” OR “Neonatal Nurses” OR “Community Health Nurse” OR “Community Health Nurses” OR “Visiting Nurses” OR “Visiting Nurse” OR “Home Nurses” OR “Home Nurse” OR “Home Health Nurses” OR “Home Health Nurse” OR “Health Visitors” OR “Health Visitor” OR “International Nurse” OR “International Nurses” OR “Foreign Nurse” OR “Foreign Nurses” OR “Male Nurse” OR “Male Nurses” OR “Public Health Nurse” OR “Public Health Nurses” OR “Nursing Staffs” OR “Hospital Nursing Staff” OR “Hospital Nursing Staffs” OR “Nutritionist” OR “Dieticians” OR “Dietician” OR “Dietitians” OR “Dietitian” OR “Occupational Therapist” OR “Optometrist” OR “Hospital Personnel” OR “Hospital Volunteer” OR “Pharmacist” OR “Clinical Pharmacists” OR “Clinical Pharmacist” OR “Physical Therapist” OR “Physiotherapists” OR “Physiotherapist” OR “Physician Executive” OR “Medical Directors” OR “Medical Director” OR “Physician” OR “Allergist” OR “Cardiologist” OR “Dermatologist” OR “Endocrinologist” OR “Foreign Medical Graduate” OR “Gastroenterologist” OR “Hepatologists” OR “Hepatologist” OR “General Practitioner” OR “General Practice Physician” OR “General Practice Physicians” OR “Geriatrician” OR “Gerontologists” OR “Gerontologist” OR “Nephrologist” OR “Neurologist” OR “Occupational Health Physician” OR “Company Physicians” OR “Company Physician” OR “Oncologist” OR “Radiation Oncologist” OR “Ophthalmologist” OR “Osteopath” OR “Osteopaths” OR “Doctor of Osteopathy” OR “Osteopathy Doctor” OR “Osteopathy Doctors” OR “Osteopathic Physician” OR “Otolaryngologist” OR “Otologists” OR “Otologist” OR “Pathologist” OR “Pediatrician” OR “Neonatologist” OR “Physiatrist” OR “Family Physician” OR “Family Physicians” OR “Primary Care Physician” OR “Primary Care Physicians” OR “Woman Physician” OR “Woman Physicians” OR “Women Physicians” OR “Pulmonologist” OR “Radiologist” OR “Rheumatologist” OR “Surgeon” OR “Barber Surgeon” OR “Neurosurgeon” OR “Neurological Surgeons” OR “Neurological Surgeon” OR “Orthopedic Surgeon” OR “Orthopedists” OR “Orthopedist” OR “Urologist” OR “Veterinarian” OR “Health Personnel” OR “Allied Health Personnel” OR “Animal Technicians” OR “Community Health Workers” OR “Dental Auxiliaries” OR “Dental Assistants” OR “Dental Hygienists” OR “Dental Technicians” OR “Denturists” OR “Emergency Medical Technicians” OR “Home Health Aides” OR “Licensed Practical Nurses” OR “Medical Record Administrators” OR “Medical Secretaries” OR “Medical Receptionists” OR “Nurses Aides” OR “Psychiatric Aides” OR “Operating Room Technicians” OR “Pharmacy Technicians” OR “Physical Therapist Assistants” OR “Physician Assistants” OR “Ophthalmic Assistants” OR “Pediatric Assistants” OR “Anatomists” OR “Anesthetists” OR “Anesthesiologists” OR “Nurse Anesthetists” OR “Audiologists” OR “Caregivers” OR “Case Managers” OR “Coroners and Medical Examiners” OR “Dental Staff” OR “Dentists” OR “Endodontists” OR “Oral and Maxillofacial Surgeons” OR “Orthodontists” OR “Doulas” OR “Emergency Medical Dispatcher” OR “Epidemiologists” OR “Health Educators” OR “Health Facility Administrators” OR “Hospital Administrators” OR “Infection Control Practitioners” OR “Medical Chaperones” OR “Medical Laboratory Personnel” OR “Medical Staff” OR “Hospitalists” OR “Nurses” OR “Nurse Administrators” OR “Nurse Practitioners” OR “Family Nurse Practitioners” OR “Pediatric Nurse Practitioners” OR “Nurse Specialists” OR “Nurse Anesthetists” OR “Nurse Clinicians” OR “Nurse Midwives” OR “Nursing Staff” OR “Nutritionists” OR “Occupational Therapists” OR “Optometrists” OR “Hospital Administrators” OR “Hospital Volunteers” OR “Hospitalists” OR “Pharmacists” OR “Physical Therapists” OR “Physician Executives” OR “Physicians” OR “Allergists” OR “Anesthesiologists” OR “Cardiologists” OR “Dermatologists” OR “Endocrinologists” OR “Foreign Medical Graduates” OR “Gastroenterologists” OR “General Practitioners” OR “Geriatricians” OR “Hospitalists” OR “Nephrologists” OR “Neurologists” OR “Occupational Health Physicians” OR “Oncologists” OR “Radiation Oncologists” OR “Ophthalmologists” OR “Osteopathic Physicians” OR “Otolaryngologists” OR “Pathologists” OR “Pediatricians” OR “Neonatologists” OR “Physiatrists” OR “Pulmonologists” OR “Radiologists” OR “Radiation Oncologists” OR “Rheumatologists” OR “Surgeons” OR “Barber Surgeons” OR “Neurosurgeons” OR “Orthopedic Surgeons” OR “Urologists” OR “Veterinarians” OR “Clinical Clerkship” OR “Pharmacy Residencies” OR “Clinical Clerkships” OR “Clinical Apprenticeship” OR “Clinical Apprenticeships” OR “Dental Education” OR “Medical Education” OR “Internship” OR “Internships” OR “Residency” OR “Residencies” OR “Nursing Education” OR “Nursing Educations” OR “Nursing Diploma Program” OR “Nursing Diploma Programs” OR “Pharmaceutical Education” OR “Pharmacy Education” OR “Pharmaceutic Education” OR “Pharmacy Residency” OR “Pharmacy Internships” OR “Pharmacy Internship” OR “Public Health Education “ OR “Veterinary Education” OR “Veterinary Educations” OR “Nonmedical Internship” OR “Nonmedical Internships” OR “Nonmedical Residencies” OR “Nonmedical Residency” OR “Non Medical Internship” OR “Non Medical Internships” OR “Medical Field Training” OR “Medical Field Studies” OR “Medical Field Study” OR “Clinical Practicum” OR “Medical Field Work” OR “Health Occupations Students” OR “Health Occupations Student” OR “Dental Students” OR “Dental Student” OR “Medical Students” OR “Medical Student” OR “Pupil Nurses” OR “Pupil Nurse” OR “Nursing Student” OR “Nursing Students” OR “Pharmacy Students” OR “Pharmacy Student” OR “Premedical Students” OR “Premedical Student” OR “Public Health Student” OR “Public Health Students” “Fellowship” OR “Fellowships” OR “Fellow” OR “Fellows” OR “healthcare education” OR “health care education” OR “health education” |
| --- |
